# Mechanisms of Mobility Control and Enhanced Oil Recovery of Weak Gels in Heterogeneous Reservoirs

**DOI:** 10.3390/gels11110854

**Published:** 2025-10-26

**Authors:** Zhengxiao Xu, Ming Sun, Lei Tao, Jiajia Bai, Wenyang Shi, Na Zhang, Yuyao Peng

**Affiliations:** 1 School of Petroleum and Natural Gas Engineering, Changzhou University, Changzhou 213164, China; ming132687@163.com (M.S.); baijiajiaa@163.com (J.B.); w.shi@cczu.edu.cn (W.S.); pyy15839421072@outlook.com (Y.P.); 2 School of Petrochemical Engineering, Changzhou University, Changzhou 213164, China; zhangna@cczu.edu.cn

**Keywords:** heterogeneity, medium–high permeability, high water cut, viscoelasticity, synergistic effect

## Abstract

At present, most oilfields in China have entered the late, high-water-cut stage, commonly facing declining single-well productivity and increasingly pronounced reservoir heterogeneity. Prolonged waterflooding has further exacerbated permeability contrast, yielding complex, hard-to-produce residual-oil distributions. Accordingly, the development of efficient enhanced oil recovery (EOR) technologies has become a strategic priority and an urgent research focus in oil and gas field development. Weak gels, typical non-Newtonian fluids, exhibit both viscous and elastic responses, and their distinctive rheology shows broad application potential for crude oil extraction in porous media. Targeting medium–high-permeability reservoirs with high water cut, this study optimized and evaluated a weak gel system. Experimental results demonstrate that the optimized weak gel system achieves remarkable oil displacement performance. The one-dimensional dual-sandpack flooding tests yielded a total recovery of 72.26%, with the weak gel flooding stage contributing an incremental recovery of 14.52%. In the physical three-dimensional model experiments, the total recovery reached 46.12%, of which the weak gel flooding phase accounted for 16.36%. Through one-dimensional sandpack flow experiments and three-dimensional physical model simulations, the oil displacement mechanisms and synergistic effects of the optimized system in heterogeneous reservoirs were systematically elucidated from macro to micro scales. The optimized system demonstrates integrated synergistic performance during flooding, effectively combining mobility control, displacement, and oil-washing mechanisms. Macroscopically, it effectively strips residual oil in high-permeability zones via viscosity enhancement and viscoelastic effects, efficiently blocks high-permeability channels, diverts flow to medium-permeability regions, and enhances macroscopic sweep efficiency. Microscopically, it mobilizes residual oil via normal stress action and a filamentous transport mechanism, improving oil-washing efficiency and increasing ultimate oil recovery. This study demonstrates the technical feasibility and practical effectiveness of the optimized weak gel system for enhancing oil recovery in heterogeneous reservoirs, providing critical technical support for the efficient development of medium–high-permeability reservoirs with high water cut.

## 1. Introduction

As many Chinese oilfields have matured into the high-water-cut stage, operators face declining single-well productivity and accentuated reservoir heterogeneity. Prolonged waterflooding exacerbates permeability contrast, redistributes residual oil into poorly swept zones, and makes capillary-trapped oil increasingly difficult to mobilize [1,2]. Consequently, the development and deployment of enhanced oil recovery (EOR) technologies have become strategic priorities in oil and gas field development. The core mechanism involves overcoming microscopic flow barriers dominated by capillary and viscous forces, thereby efficiently mobilizing residual oil and improving ultimate recovery [3,4,5,6]. Conventional water flooding, while being an economically viable primary development method in early stages, leads to progressive reservoir degradation with continued injection [7,8]. The process intensifies reservoir heterogeneity, amplifies permeability contrasts, and may induce formation damage [9,10,11].

Polymer flooding is a relatively well-established and widely applied chemical EOR method [12]. It involves dissolving water-soluble polymers—such as hydrolyzed polyacrylamide (HPAM), hydrophobically associating polymers, and polyacrylamide-based copolymers—in the injected water [13,14,15,16,17]. The principal mechanisms are improvement in the oil–water mobility ratio and enhancement in sweep efficiency at both macroscopic and microscopic scales [18,19,20,21,22]. Weak gel flooding [5,23,24,25,26] relies fundamentally on the use of crosslinkers to bridge polymer molecular chains, forming a three-dimensional network with excellent viscoelasticity and structural strength. At the macroscopic scale, mobility control is achieved by increasing the viscosity of the aqueous phase; at the pore scale, elastic displacement is critical, whereby stored and released elastic energy effectively mobilizes residual oil, lowers residual oil saturation, and improves overall displacement efficiency, thereby providing an effective solution for oilfield development [27,28,29,30]. However, under complex reservoir conditions, weak gel systems that are not tailored to specific reservoir properties may yield displacement performance far below expectations or even negative outcomes. In heterogeneous reservoirs, poorly matched rheological parameters can undermine mobility control, leaving low-permeability zones unswept and causing channeling through high-permeability zones, thereby exacerbating interlayer conflicts [31,32,33]. In homogeneous reservoirs, excessively high viscoelasticity can sharply increase injection pressure, induce in situ shear degradation, and lead to severe energy loss [34].

Current research has clarified the mechanisms of macroscopic mobility control and microscopic displacement by weak gels, with notable advances in improving sweep efficiency at both scales. Ali H. S. et al. [35], through a review of project cases, reported a strong correlation between optimal polymer viscosity and both oil viscosity and permeability contrast, emphasizing that matching polymer viscosity to specific reservoir parameters is essential for successful polymer flooding. Alisheva Z. et al. [36] experimentally demonstrated the interaction between polymer viscoelasticity and wettability: under strongly water-wet conditions, elasticity enhances oil displacement efficiency, whereas in weakly water-wet systems, it inhibits oil droplet deformation; moreover, the direction of normal stress changes with increasing elasticity. Xuwei L. et al. [37] employed numerical simulation and the response surface method to optimize polymer selection, concluding that low-viscosity polymer solutions with a high residual resistance factor (RRF) are more suitable for heterogeneous heavy-oil reservoirs. Di Q et al. [38], based on nuclear magnetic resonance (NMR) imaging studies, found that weak gels exhibit an integrated migration pattern during subsequent water flooding processes and that different polymer-weak gel displacement modes have a decisive influence on the distribution of residual oil within core samples. Hatzignatiou G D et al. [39] conducted experimental screening and evaluation of various polymers and weak gels, investigating the effects of parameters such as filterability, injectivity, gelation time, gel strength, and gel shrinkage ratio on oil recovery. The study demonstrated that the Type A gel exhibited excellent stability, high strength, and the ability to withstand the pressures exerted during subsequent water flooding. Yajuan T et al. [40] developed a temperature-tolerant weak gel system suitable for high-salinity injection water environments to address challenges associated with highly mineralized injection fluids. Through dual-sandpack parallel oil displacement experiments, the study verified that this weak gel system exhibited excellent migration capability and plugging performance.

Although recent research has advanced weak gel flooding, the dynamic interaction between weak gels, complex pore structures, and fluid distributions in three-dimensional physical simulations, particularly those representative of heterogeneous reservoirs, remains insufficiently characterized. Against this backdrop, this study targets medium–high-permeability reservoirs with high water cut. A physical three-dimensional model was constructed to reproduce the target reservoir properties, and the viscosities of weak gel solutions at different concentrations were measured to optimize a formulation suited to the reservoir conditions. Through dual-sandpack flow experiments and physical three-dimensional model displacement tests, as well as by integrating macroscopic displacement performance, pressure data, and fractional-flow behavior, we comprehensively evaluated the displacement efficiency of the optimized weak gel system. Furthermore, depth-of-field microscopy and scanning electron microscopy (SEM) were used to analyze, at the pore scale, the weak gel displacement mechanism and the mobilization of residual oil. The optimized system exhibits a synergistic effect during flooding by integrating mobility control, displacement, and oil-washing mechanisms. At the macroscopic scale, it enhances fluid viscosity and exerts viscoelastic effects to efficiently displace residual oil in high-permeability zones while selectively plugging high-permeability channels to divert fluid into medium-permeability regions, thereby improving macroscopic sweep efficiency. At the microscopic scale, the system mobilizes residual oil through normal stress effects and a filamentous transport mechanism, enhancing oil-washing efficiency and ultimately increasing final recovery. This study provides a practical and theoretical basis for enhancing oil recovery using optimized weak gel systems in medium–high-permeability, high-water-cut reservoirs.

## 2. Results and Discussion

### 2.1. Optimization of Polymer Concentration in Weak Gel System

**Figure 1:**
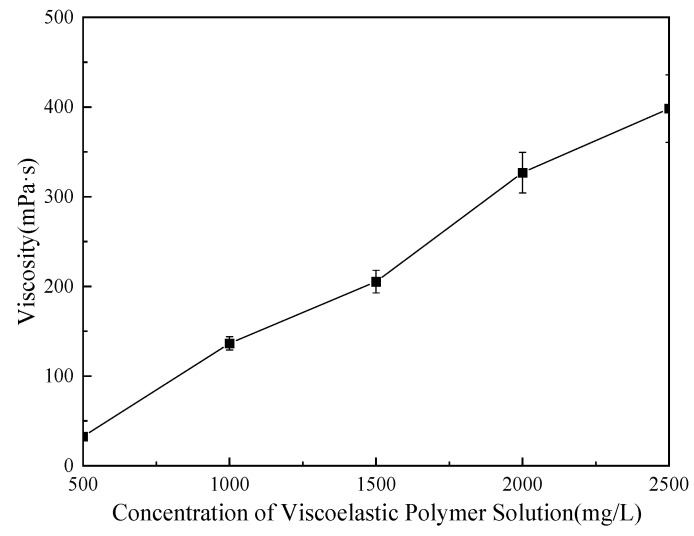
Viscosity curve of the weak gel at different concentrations.

Given the measured crude oil viscosity of 37.2 mPa·s at 60 °C in the target reservoir, this study optimized the weak gel system by evaluating five concentrations: 500, 1000, 1500, 2000, and 2500 mg/L. Viscosities of the polymer solutions were measured using an Anton Paar MCR 302 rheometer, and the resulting viscosity curves for each concentration are shown in Figure 1. The optimal concentration was selected based on the ratio of polymer solution viscosity to crude oil viscosity under reservoir conditions. This approach ensures effective mobility control and is expected to contribute to enhanced oil recovery performance.

As shown in Figure 1, the polymer solution viscosity increases approximately linearly with concentration over the tested range (500–2500 mg/L). According to established guidelines for the polymer-to-crude viscosity ratio [41,42], the target crude is classified as medium–high viscosity, for which an optimal ratio of 2:1–4:1 is recommended. At a polymer concentration of 1000 mg/L, the measured viscosity is 137 mPa·s, about 3.68 times that of the crude oil, meeting the target range; therefore, 1000 mg/L was selected as the optimal concentration for the reservoir conditions. A trace amount of crosslinker was then added, and the mixture was stirred at 700 r/min for 1 h. After preparation, the system was aged for 24 h to obtain the optimized weak gel system.

### 2.2. Dual-Sandpack Weak Gel Flooding Experiment

**Figure 2:**
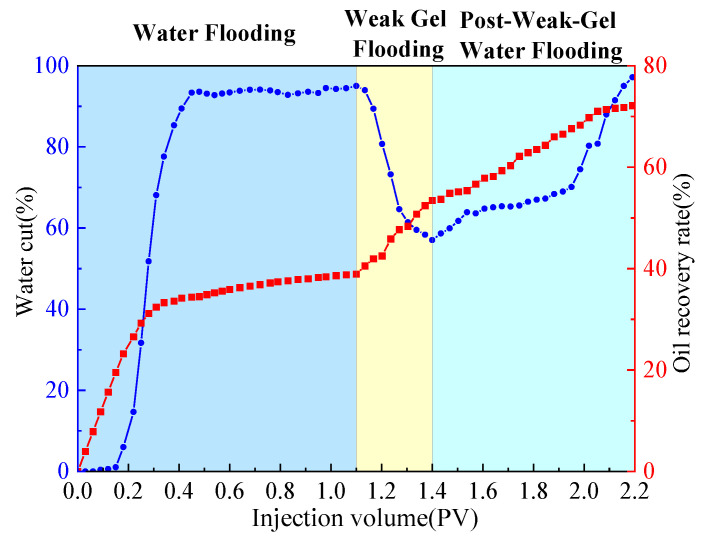
Water cut and recovery rate during weak gel flooding in dual-sandpack models.

**Figure 3:**
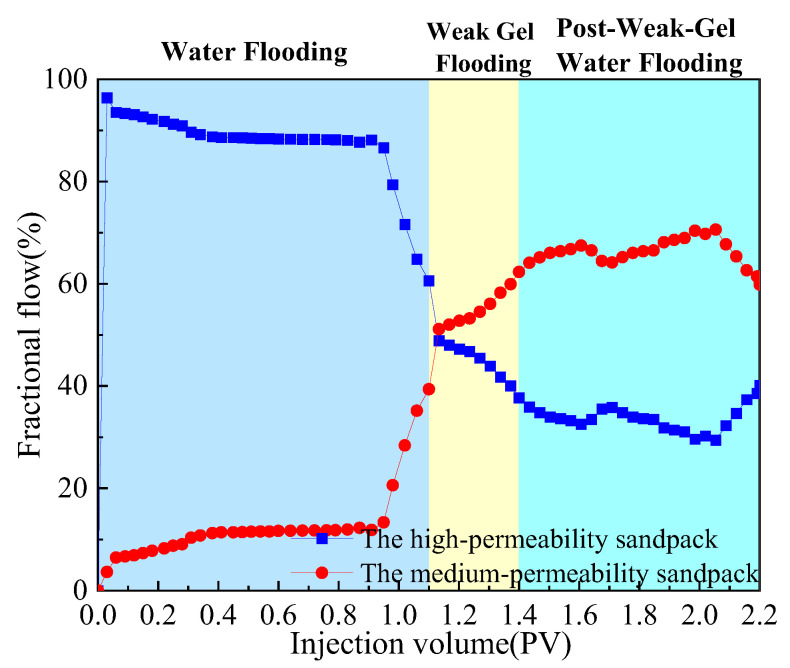
Fractional flow from both sandpacks during weak gel flooding in dual-sandpack models.

**Figure 4:**
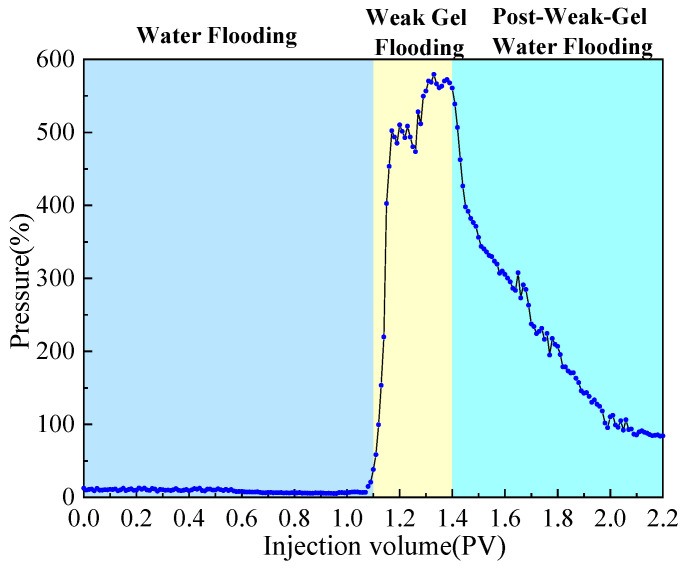
Injection–production differential pressure during weak gel flooding in the dual-sandpack models.

The dual-sandpack models, pre-saturated with oil and water, were connected in parallel and subjected to the following sequential flooding process: First, water flooding was conducted at a flow rate of 1 mL/min until the water cut exceeded 90%. This was followed by the injection of 0.3 PV of a weak gel solution at a concentration of 1000 mg/L while maintaining the flow rate at 1 mL/min. Finally, water flooding was resumed at the same flow rate until the water cut reached approximately 95%, marking the end of the experiment. Throughout the experiment, the volumes of oil and water produced from both sandpacks were measured in 10 min intervals, with graduated cylinders being replaced promptly after each measurement. Based on these measurements, water cut, oil recovery, and fractional flow were calculated and are plotted in Figure 2 and Figure 3. Meanwhile, pressure sensors were used to acquire the injection–production differential pressure data once per second. The collected pressure data were processed to generate the differential pressure curve presented in Figure 4.

As shown comprehensively in Figure 2, Figure 3 and Figure 4, the oil recovery rate during the water flooding stage was 38.91%, reflecting a significant imbalance in the displacement efficiency between the medium- and high-permeability zones under high-water-cut conditions. During this stage, the injection–production differential pressure remained essentially stable. The water cut increased rapidly in the initial phase and stabilized around 0.4 PV of fluid injected, while the rate of increase in oil recovery became slower. Fractional-flow trends indicate that the injected fluid initially entered the high-permeability sandpack, after which the fractional flow from this unit gradually decreased. Upon weak gel injection, the viscoelastic solution preferentially accessed high-permeability channels, impeding the preferential water flow paths. Owing to viscoelastic deformation, additional normal stresses were generated, producing a microscopic, selective, water-blocking, oil-promoting effect [6,23]. These stresses enabled displacement of previously unswept oil in the medium-permeability zones through pore throats, mobilizing residual oil in the medium-permeability sandpack, which experimentally manifested as a sustained increase in flow from this sandpack at later times. The underlying mechanism is effective plugging and mobility control by the weak gel via viscoelastic effects and physical retention.

In the subsequent water-flooding stage, an additional 18.83% incremental oil recovery was achieved, although the rate of increase was lower than during weak gel flooding. The injection–production differential pressure declined, while the water cut rose initially, then decreased, and ultimately increased again. The fractional flow from the medium-permeability sandpack first increased and then slightly decreased. These results indicate that weak gel flooding not only significantly increased production but also modified the distribution of the remaining oil, creating a more balanced displacement environment for the subsequent waterflood. This further confirms that the adsorption and retention of the weak gel in pore channels provided sustained benefits. In conclusion, within the one-dimensional dual-sandpack model, the weak gel significantly enhanced oil recovery by improving mobility control, increasing sweep efficiency in medium-permeability zones, and effectively mobilizing residual oil.

### 2.3. Physical Three-Dimensional Model Experiment of Weak Gel Flooding

#### 2.3.1. Analysis of Experimental Results from Physical Three-Dimensional Model

**Figure 5:**
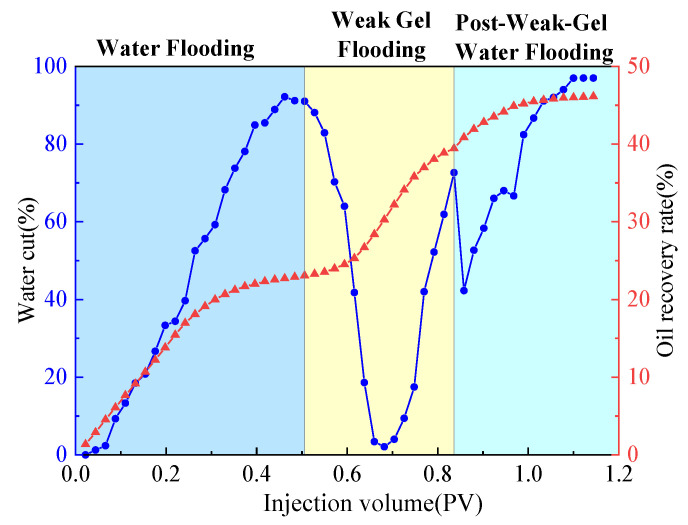
Variation in water cut and oil recovery rate during weak gel flooding in physical three-dimensional model.

**Figure 6:**
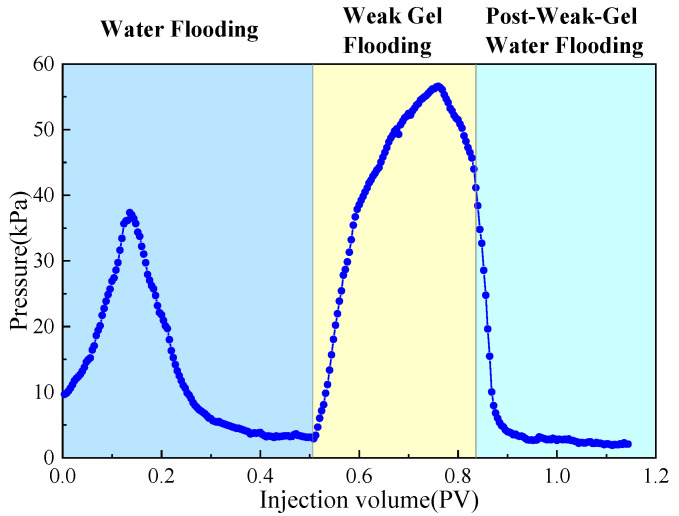
Variation in injection–production differential pressure during weak gel flooding in physical three-dimensional model.

**Figure 7:**
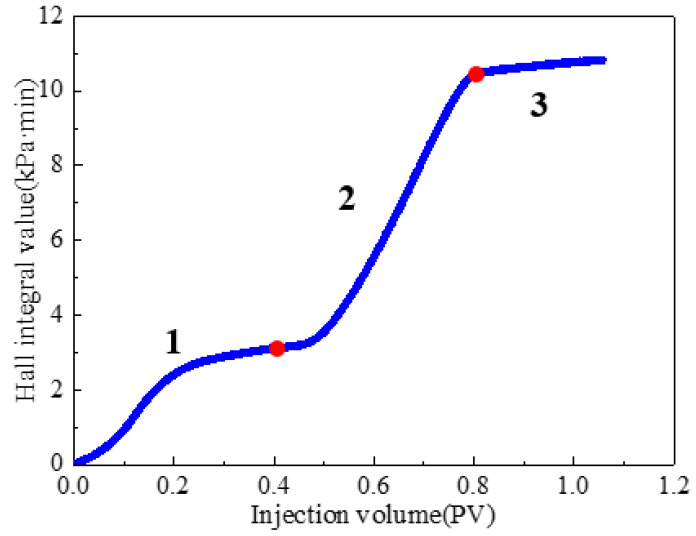
Hall plot for weak gel flooding in physical three-dimensional model.

A three-dimensional physical model was constructed to simulate the target reservoir’s geological characteristics. The injection–production pattern employed one injection well and one production well, positioned at diagonal corners of the model, each located 5 cm from the adjacent boundaries. Permeability was distributed with the upper layer having a permeability of 500 mD and that of the lower layer was 1500 mD. The experimental process consisted of the following stages: (1) Water flooding: brine was injected at a constant rate of 10 mL/min until the water cut exceeded 90%. (2) Weak gel flooding: a weak gel solution with a concentration of 1000 mg/L was injected at 0.3 PV and a rate of 10 mL/min. (3) Post-weak-gel water flooding: brine injection was resumed at 10 mL/min until the water cut reached approximately 95%, marking the end of the experiment. During the experiment, the oil and water production volumes at the outlet of the physical three-dimensional model were recorded every 10 min, with graduated cylinders being replaced promptly after each measurement. After the experiment, the data were processed to calculate the water cut and oil recovery rate, and the results are presented in Figure 5. Differential pressure data were recorded by pressure sensors in 1 s intervals, and the injection–production pressure difference curve is plotted in Figure 6. Based on parameters such as injection volume, pressure, and injection time, a Hall plot was generated and is presented in Figure 7.

As shown in Figure 5 and Figure 6, the cumulative oil recovery reached 46.12%. Analysis of the Hall plot (Figure 7) indicates that during Segment 1 (water flooding) recovery was 23.08%; the Hall plot rose initially and then approached a steady slope, accompanied by a rapid increase in water cut and a brief pressure rise before stabilization. During Segment 2 (weak gel flooding), recovery reached 16.36%; the water cut displayed a V-shaped trajectory with a minimum of 2.08%. The injection–production pressure increased rapidly before this minimum and then declined, indicating that the weak gel initially entered and partially plugged high-permeability channels, diverting flow to medium-permeability zones until weak gel breakthrough and/or shear-thinning reduced flow resistance and redirected flow toward high-permeability paths. In Segment 3 (post-weak-gel water flooding), an additional 6.68% was recovered; the water cut rose rapidly but at a decreasing rate and pressure remained stable, suggesting that the weak gel flood had already mobilized most of the remaining oil, leaving limited potential for further production.

#### 2.3.2. Analysis of Oil Displacement Efficiency in Physical Three-Dimensional Model

**Figure 8:**
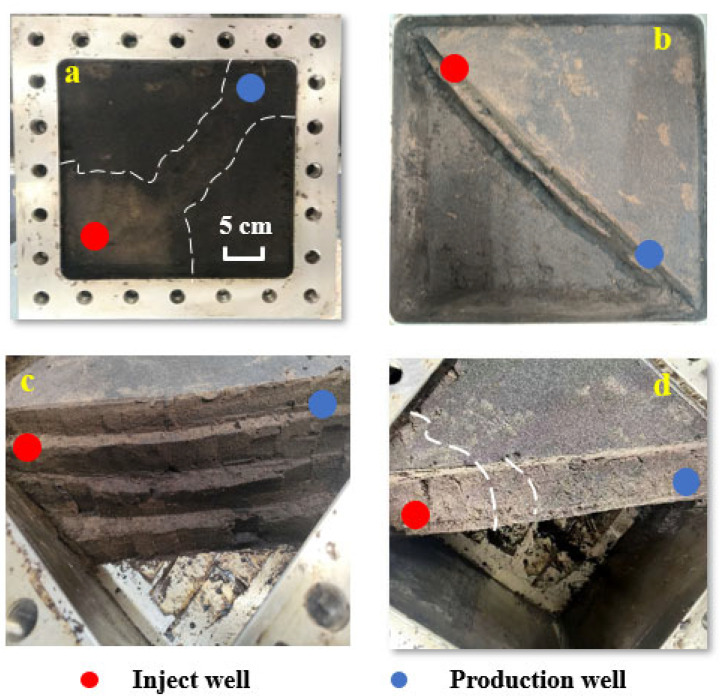
Actual oil displacement effect of weak gel flooding in physical three-dimensional model: (**a**–**d**) present the four stages of the post-flooding excavation process from the three-dimensional physical model after weak gel flooding.

Following the weak gel flooding in the physical three-dimensional model, the model was opened for post-test visual inspection to assess displacement performance. As shown in Figure 8, a significant oil displacement effect was observed particularly near the injection and production wells, with dashed lines in the figure delineating the sweep behavior of the weak gel flooding. A distinct flow channel formed by the weak gel was clearly visible between the two wells, indicating that the optimized weak gel system exhibited high oil-washing efficiency and strong sweep capability.

**Figure 9:**
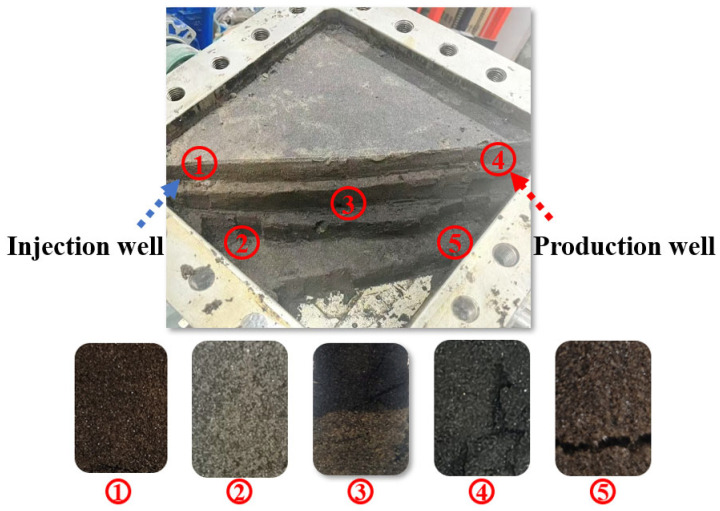
Sampling distribution for post-flooding analysis in physical 3D model of weak gel flooding.

Post-flood sampling was conducted to assess displacement effectiveness in the physical three-dimensional model. Five sand-layer samples were collected along the line between the injection and production wells (Figure 9), specifically taken from the medium-permeability zone near the injection well, the high-permeability zone near the injection well, the transitional medium–high permeability zone at the midpoint between the injection and production wells, the medium-permeability zone near the production well, and the central high-permeability zone near the production well. 

This sampling strategy enabled a comprehensive analysis of crude oil mobilization and viscoelastic weak gel migration across permeability layers and spatial regions during flooding, providing critical insights into displacement characteristics and fluid propagation behavior in heterogeneous reservoirs.

#### 2.3.3. Microscopic Residual Oil Analysis and Post-Flooding Scanning Electron Microscopy (SEM) Characterization

**Figure 10:**
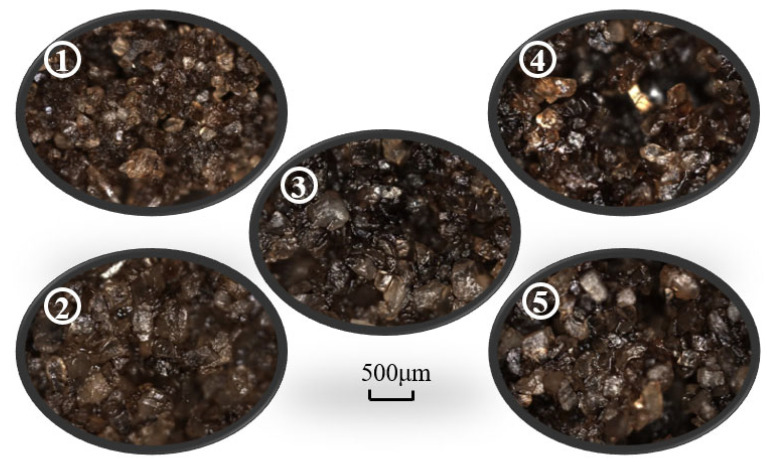
Depth-of-field microscopic analysis of physical 3D Model samples after weak gel flooding.

**Figure 11:**
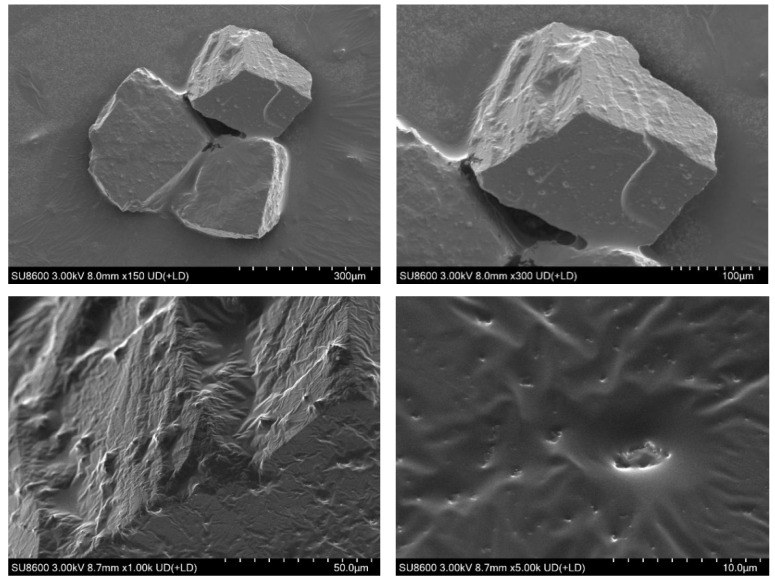
SEM images of the samples.

The five sand samples shown in Figure 9 were examined with a depth-of-field microscope; the resulting micrographs are shown in Figure 10. The sample from the high-permeability zone near the production well was air-dried and subsequently analyzed by scanning electron microscopy (SEM) to characterize the weak gel’s microscopic morphology. The SEM results are presented in Figure 11.

Based on the depth-of-field micrographs and SEM images, together with the observed displacement effects during sampling, the following conclusions were drawn: the medium-permeability layers exhibited higher residual-oil saturation with lower moisture, whereas the high-permeability zones showed markedly higher displacement efficiency with greater moisture and visible residual weak gel. These observations indicate that the weak gel preferentially entered and blocked high-permeability channels, mobilizing movable residual oil along these pathways. During the water-flooding stage, the injected fluid primarily swept the high-permeability zones, resulting in limited coverage of the upper medium-permeability regions.

In the SEM images magnified at 5000× (Figure 11), the sand particle surfaces appear smooth-textured overall, lacking the rough crystalline structures typical of mineral grains. This suggested that the original surfaces were covered by a residual layer. Further observation revealed that this coating exhibited distinct “flow-induced elongated textures” and wrinkled membrane-like morphologies, which were identified as residual film structures formed by the weak gel and crude oil remaining on the particle surfaces after the flooding process.

In the center of the image, a concave feature approximately 2–3 μm in size is observed, with its margins showing a distinct edge-brightening (“highlighted edges”) effect, likely due to charge accumulation caused by electron-beam interaction with agglomerated weak gel. This observation further supports the presence of residual weak gel on the sand grain surface. Taken together with the surface morphology, namely, the wrinkled, membrane-like structures and highlighted edges, it was preliminarily concluded that an oil film or a mixed oil/viscoelastic weak gel residual layer existed on the sample surface. These morphological characteristics suggest that the weak gel underwent flow deposition during displacement, which, while enhancing oil recovery efficiency, may have also contributed to localized entrapment of residual oil.

Based on the experimental data, Hall plot analysis, depth-of-field microscopy, and SEM imaging, the mechanisms of residual oil mobilization by viscoelastic weak gel flooding are summarized as follows: Mobilization arises from mobility control coupled with physicochemical synergistic effects. The weak gel’s viscoelasticity and the additional normal stresses generated by extensional deformation promote displacement of residual oil in high-permeability zones and, to a lesser extent, in medium-permeability zones. This establishes flow barriers that force fluid diversion and produce macroscopic fluid redistribution. At the pore scale, elastic effects and normal stresses disturb the equilibrium at the oil–water–rock three-phase interface, mobilizing additional residual oil. The weak gel exhibits shear thinning and extensional thickening behavior: the lower apparent viscosity in high-permeability channels enhances penetration and plugging efficiency, whereas higher apparent viscosity in medium-permeability zones increases displacement pressure [8,26]. This dual rheology enables the weak gel to enter large pores to impose flow resistance while displacing residual oil from small pores, thereby improving microscopic sweep efficiency [43,44]. The weak gel thus enables multiscale mobilization: macroscopically, it balances sweep coverage; microscopically, it expels residual oil from blind pores via elastic deformation, reduces oil–water interfacial tension, promotes crude-oil emulsification, and mobilizes dispersed residual oil through filamentous stretching. The wrinkled residual weak gel observed in high-permeability zones evidences flow deposition that forms microscopic barriers, ultimately enhancing oil recovery.

In summary, the oil recovery rates at various stages calculated from the dual-sandpack experiments and three-dimensional physical simulation tests are presented in Table 1.

## 3. Conclusions

The dual-sandpack results indicate that the weak gel (concentration: 1000 mg/L) flooding stage yielded an additional 14.52% oil recovery, and the subsequent waterflooding stage contributed a further 18.83%, demonstrating strong displacement performance and sustained EOR effects. By increasing aqueous phase viscosity and reducing the oil–water mobility ratio, the weak gel preferentially entered and blocked high-permeability channels, diverting flow into the medium-permeability sandpack and thereby improving sweep efficiency. At the pore scale, normal-stress effects effectively mobilized residual oil, with physical retention and chemical adsorption acting synergistically.

The physical three-dimensional model flooding experiment systematically evaluated the displacement performance and sweep behavior of a weak gel (concentration: 1000 mg/L) in a heterogeneous reservoir. The weak-gel-flooding stage increased oil recovery by 16.36%, with an additional 6.68% obtained during subsequent waterflooding. Macroscopically, a distinct flow channel developed between the injector and producer, corroborated by clear color contrasts in the sand layers, indicating differential oil mobilization. Microscopically, normal-stress and viscoelastic effects displaced residual oil from medium- and high-permeability zones by establishing a mobility-control barrier that enhanced fluid diversion. Through flow-deposition, the weak gel formed microscopic plugs, reduced oil–water interfacial tension, promoted crude oil emulsification, and mobilized and transported dispersed residual oil via filamentous displacement, collectively improving oil-washing efficiency.

This study elucidates, at both macroscopic and microscopic scales, the displacement mechanisms and synergistic effects of the optimized weak gel system in heterogeneous reservoirs. The system integrates three coupled functions, mobility control, oil displacement, and oil washing, demonstrating its feasibility and effectiveness for enhancing oil recovery in heterogeneous formations. The results provide reliable technical support for improving recovery in medium–high-permeability heterogeneous reservoirs with high water cut.

## 4. Materials and Methods

### 4.1. Materials and Equipment

The synthetic formation water was prepared to match the ionic composition of field brine from the target reservoir, with per-liter ion concentrations listed in Table 2. Three grades of quartz sand, 60–80 mesh, 80–100 mesh, and 100–120 mesh, were employed to simulate the reservoir geology. These sands were compacted and packed to construct both dual-sandpack models and a physical three-dimensional model with a positive rhythmic permeability distribution. The dual-sandpack models exhibited permeabilities of 500 mD and 1500 mD, while the physical three-dimensional model was designed with an upper-layer permeability of 500 mD and a lower-layer permeability of 1500 mD. Each sandpack model measured 2.5 cm in diameter and 60 cm in length, with a maximum pressure tolerance of 30 MPa. The physical three-dimensional model featured an internal chamber measuring 30 cm × 30 cm × 20 cm, capable of withstanding pressures up to 10 MPa and temperatures up to 80 °C. The model incorporated multiple integrated monitoring points for real-time data acquisition, meeting the experimental requirements.

Experimental instruments comprised an electric stirrer; an MCR 302 rheometer (Anton Paar, Graz, Austria); an OHR-M2G precision pressure sensor (HORIBA, Ltd., Kyoto, Japan); an ISCO dual-plunger metering pump (Model 100DX, IL, Tridan, Danville, IL, USA) for injecting the aqueous urea solution; a depth-of-field microscope DVM2500 (Leica Microsystems, Wetzlar, Germany); and a scanning electron microscope GeminiSEM 450 (Carl Zeiss Microscopy GmbH, Oberkochen, Germany).

### 4.2. Optimization of Polymer Concentration 

Using the pre-optimized concentration appropriate for the reservoir conditions as a baseline, viscosities of polymer solutions at multiple concentrations were systematically measured, the optimal concentration was identified, and a weak gel system tailored to the target reservoir was formulated.

#### 4.2.1. Preparation of Polymer Solutions

A 5000 mg L^−1^ weak gel stock solution was prepared by dissolving 2.5 g of the oil-displacing agent in 497.5 g of distilled water. Using a magnetic stirrer initially set to 400 r/min, the polymer was added gradually along the vortex wall; the stirring speed was then increased to 700 r/min and maintained for 1 h to ensure complete dissolution. The stock solution was allowed to stand for 24 h. It was subsequently diluted with synthetic formation water to target concentrations of 500, 1000, 1500, 2000, and 2500 mg/L. During dilution, the mixture was stirred continuously at 300 r min^−1^ for 15 min to obtain the target weak gel solutions.

#### 4.2.2. Viscosity Measurement of Weak Gel Solutions at Various Concentrations

The measuring rotor of the Anton Paar MCR 302 rheometer was installed by aligning the vertical grooves on the rotor tip with those on the instrument interface. After mounting, torque, angle, and temperature calibrations were performed. Using a pipette, the solution was carefully loaded into the center of the fixture to avoid air bubbles. Silicone oil or a solvent trap was applied to prevent evaporation. The upper fixture was gradually lowered to the preset gap. Prior to each sample measurement, the fixture was thoroughly cleaned and dried with an appropriate solvent. The measurement was conducted at 60 °C with a shear rate of 7.34 s^−1^ over a duration of 3 min. Thirty data points were recorded for each weak gel solution. The viscosity values at different concentrations (500, 1000, 1500, 2000, and 2500 mg/L) were collected to establish the relationship between polymer concentration and viscosity. The discrete distribution of viscosity values was assessed by calculating the measurement error. A trace amount of crosslinker was then added, and the mixture was stirred and allowed to stand, resulting in the formation of the optimized weak gel system.

### 4.3. Weak-Gel-Flooding Sandpack Experiment

**Figure 12:**
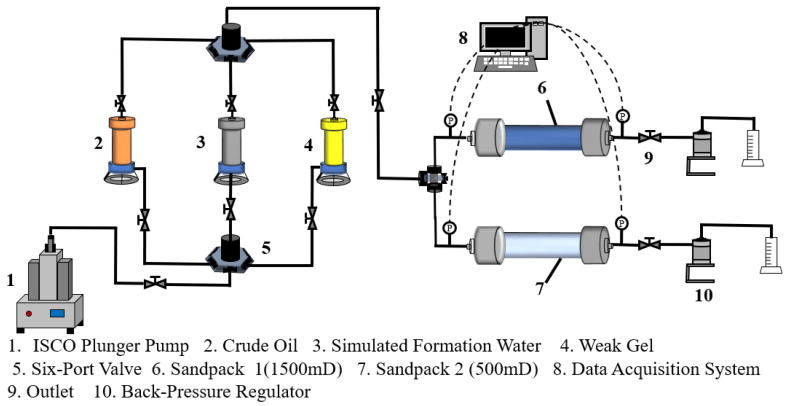
The dual-sandpack oil displacement experiment.

Dual-sandpack models with permeabilities of 500 mD and 1500 mD were prepared and connected in parallel (schematic shown in Figure 12). After packing with quartz sand, the models were vacuumed and sequentially saturated with brine and crude oil. The experimental procedure comprised the following stages: (1) Water flooding: brine was injected at a constant rate of 1.0 mL/min until the water cut exceeded 90%. (2) Weak gel flooding: a weak gel solution (1000 mg/L concentration) was injected at 0.3 PV and a rate of 1.0 mL/min. (3) Post-weak-gel water flooding: brine injection was resumed at 1.0 mL/min until the water cut reached approximately 95%, marking the end of the experiment. The produced fluid at the outlet was visually observed, and experimental data (including production rates and differential pressures) were recorded. Subsequently, the data were systematically analyzed to evaluate the oil displacement performance of the weak gel and investigate the mechanisms of residual oil mobilization.

### 4.4. Physical Three-Dimensional Model Experiment 

**Figure 13:**
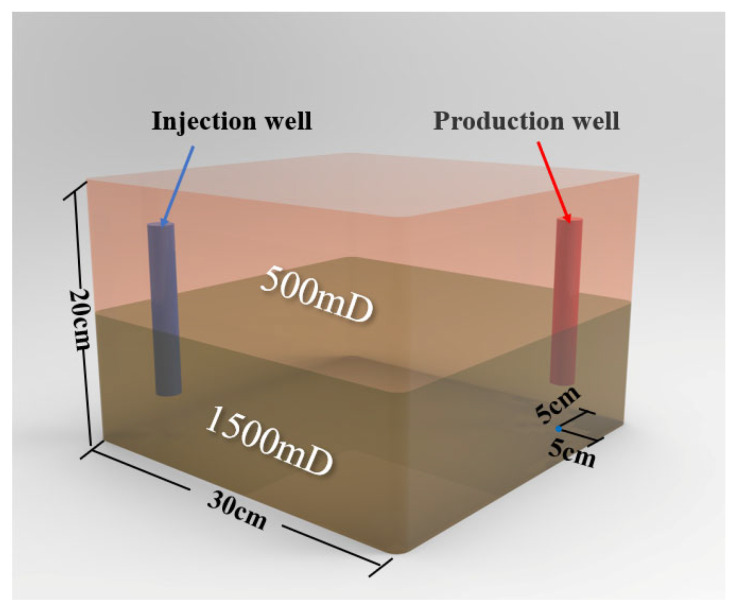
The well configuration and permeability distribution in the physical three-dimensional model.

**Figure 14:**
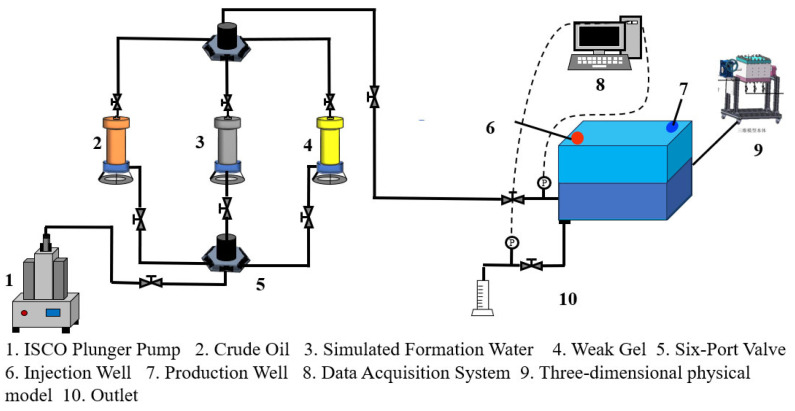
The oil displacement process in the physical three-dimensional model.

A physical three-dimensional model was constructed to simulate the target reservoir’s geological characteristics. The injection–production pattern employed one injection well and one production well, positioned at diagonal corners of the model, each located 5 cm from the adjacent boundaries. Permeability was distributed in a positive rhythmic pattern (Figure 13), with the upper layer having a permeability of 500 mD and that of the lower layer being 1500 mD. Quartz sand was proportionally packed and compacted layer-by-layer to ensure uniform density and formation homogeneity. After packing, the model was sealed with a cover plate and securely fastened to guarantee overall containment. The model was subsequently saturated with brine and crude oil. A schematic of the experimental procedure is provided in Figure 14. The experimental process consisted of the following stages. (1) Water flooding: brine was injected at a constant rate of 10 mL/min until the water cut exceeded 90%. (2) Weak gel flooding: a weak gel solution with a concentration of 1000 mg/L was injected at 0.3 PV and a rate of 10 mL/min. (3) Post-Weak-gel water flooding: brine injection was resumed at 10 mL/min until the water cut reached approximately 95%, marking the end of the experiment.

## Figures and Tables

**Table 1:** Comparison of oil recovery rates from weak gel flooding experiments in dual-sandpack and physical three-dimensional models.

Model Type	Water-Flooding Stage %	Weak-Gel-Flooding Stage %	Post-Weak-Gel Water-Flooding Stage %
Dual-Sandpack Experiment	38.91	14.52	18.83
Physical Three-Dimensional Model Experiment	23.08	16.36	6.68

**Table 2:** Ionic composition of formation water.

Component	Na_2_SO_4_	NaCl	CaCl_2_	KCl	MgCl_2_·H_2_O
**Quality (g)**	9.976	10.582	0.419	0.183	1.506

## Data Availability

The data presented in this study are available on request from the corresponding author.
